# Interictal plasma glutamate levels are elevated in individuals with episodic and chronic migraine

**DOI:** 10.1038/s41598-022-10883-9

**Published:** 2022-04-28

**Authors:** Chae Gyu Park, Min Kyung Chu

**Affiliations:** 1 Therapeutic Antibody Research Center, Genuv Inc., Seoul, Republic of Korea; 2 Laboratory of Immunology, Severance Biomedical Science Institute, Yonsei University College of Medicine, Seoul, Republic of Korea; 3 Institute for Immunology and Immunological Diseases, Yonsei University College of Medicine, Seoul, Republic of Korea; 4 Department of Life Science, Heart-Immune-Brain Network Research Center, Ewha Womans University, Seoul, Republic of Korea; 5 Department of Neurology, Severance Hospital, Yonsei University College of Medicine, 50-1 Yonsei-ro, Seodaemun-gu, Seoul, 03722 Republic of Korea

**Keywords:** Neuroscience, Neurology

## Abstract

Glutamate is implicated in migraine pathogenesis including central sensitization and pain transmission. Altered plasma glutamate levels has been noted in migraine. Chronic migraine (CM) presented a higher degree of central sensitization and pain transmission than episodic migraine (EM). However, no study has evaluated plasma glutamate levels separately in EM and CM. This study aimed to assess plasma glutamate levels in EM and CM compared to controls. An enzyme-linked immunosorbent assay was used to assess plasma glutamate levels in females with EM (n = 98) and CM (n = 92) as well as controls (n = 50). Plasma glutamate levels in participants with EM (median and interquartile range, 49.73 [40.82–66.12] μmol/L, *p* < 0.001) and CM (58.70 [44.64–72.46] μmol/L, *p* < 0.001) were significantly higher than those in controls (38.79 [29.50–53.60] μmol/L). Glutamate levels were not significantly different between participants with EM and CM (*p* = 0.075). There was no significant association of plasma glutamate levels with headache frequency (exponential and 95% confidence interval, 1.285 [0.941–1.755]) and intensity (mild, 59.95 [59.95–59.95] μmol/L vs. moderate, 52.76 [40.83–106.89] μmol/L vs. severe, 55.16 [42.34–68.03] μmol/L, *p* = 0.472). The plasma glutamate level is a potential indicator for EM and CM.

## Introduction

Glutamate is an excitatory neurotransmitter expressed abundantly in humans and is crucially involved in the pathogenesis of migraine, including central sensitization, cortical spreading depression, and trigeminal activation^1^. It is synthesized from glutamine and glucose, and is metabolized into gamma-aminobutyric acid, which is a key inhibitory neurotransmitter^2^. Anti-glutamatergic drugs are used as preventive treatments for migraine^3,4^.

During ictal and interictal periods, individuals with migraine present with altered glutamate levels in body fluids, including plasma, cerebral spinal fluid (CSF), urine, and saliva^5–12^. Many studies have noted elevated plasma glutamate levels during the interictal period. Furthermore, a meta-analysis using data pooled from previous studies found a significant elevation in interictal plasma levels of glutamate in individuals with migraine^13^.

Migraine can be categorized as episodic migraine (EM) and chronic migraine (CM)^14^. These migraine subtypes have different prevalence, comorbidities, and treatment response^15^. A previous study reported differences in the biomarkers for EM and CM^13^. Compared with individuals with EM, individuals with CM reported a higher degree of central sensitization and pain transmission^16^. Considering that glutamate plays a key role in central sensitization and pain transmission, there is a possibility that the plasma glutamate levels in CM differ from those in EM^1^. However, to our knowledge, no study has evaluated plasma glutamate levels separately in individuals with EM and CM.

We hypothesized that compared with healthy controls, individuals with EM and CM have increased interictal plasma levels of glutamate. This study aimed to assess plasma glutamate levels in participants with EM and CM and compare them with those in controls. We also aimed to assess the difference in the plasma glutamate levels between EM and CM. Additionally, the present study aimed to evaluate the diagnostic utility of plasma glutamate levels in differentiating participants with EM and CM from healthy controls. Furthermore, we aimed to investigate the relationship of plasma glutamate levels with clinical characteristics and comorbidities.

## Methods

### Participants

We recruited females with EM and CM (age: 19–65 years) who visited an outpatient clinic of the department of neurology of a tertiary university hospital between October 2019 and December 2020. The inclusion criteria were as follows: female, 19–65 years old, fulfilling the criteria specified by the third edition of the International Classification of Headache Disorders (ICHD-3) for EM (code 1.1 or 1.2) and CM (code 1.3)^14^, ≥ 48 h having passed after the cessation of a typical migraine attack, being headache-free (for participants with EM), and having mild or less headache intensity (for participants with CM). In case the participants with EM or CM were taking preventive medications, we only enrolled participants who were on stable drug dosage for ≥ 1 month as well as those who fully understood the study protocol. The exclusion criteria were as follows: having secondary headache(s) other than medication-overuse headache (MOH, code 8.2) according to the ICHD-3; having chronic pain conditions other than fibromyalgia (FM), which was diagnosed according to the 2016 American College of Rheumatology (ACR) criteria^17^; and undergoing medical or psychiatric treatments. Healthy controls were enrolled through advertisement and were considered acceptable if they had not experienced any type of headache during the previous year and had not experienced migraine attacks during their lifetime.

### Plasma collection

Blood samples were collected from the right antecubital vein between 9:00 am and 12:00 p.m. after ≥ 15 min rest. To avoid the effects of dietary glutamate on our findings, the blood sampling was performed after ≥ 2 h fasting. After centrifugation at 3000 rpm for 15 min at 4 °C, plasma was harvested and saved at − 70 °C until subsequent use.

### Measurement of plasma glutamate levels

Plasma glutamate levels were quantified using an enzyme-linked immunosorbent assay (ELISA) kit (KA1909, Lot No. 200595) (Abnova, Taipei, Taiwan) according to the manufacturer’s instructions. The minimum detection limit of the ELISA kit was 1.156 μmol/L. The estimated inter-assay and intra-assay accuracies were 4.21% and 4.11% coefficient of variation, respectively. The estimated inter-assay and intra-assay precisions were 5.09% and 6.43% coefficient of variation, respectively. The aforementioned values were calculated by an independent laboratory blinded to the clinical data.

### Estimation of the sample size

The sample size was estimated based on a previous study on the interictal level of plasma glutamate in individuals with migraine^7^. The ratio of participants with migraine to controls was set at 2:1. Assuming a 5% significance level and 80% power, the sample sizes of the migraine and control groups were calculated as 89 and 45, respectively. Accordingly, we targeted to enroll 90 participants with each migraine type (EM and CM). Furthermore, we targeted to enroll 45 participants as healthy controls.

### Assessment of anxiety, depression, FM, and medication overuse

Anxiety and depression were examined since they are common comorbidities of migraine and are closely associated with the severity of migraine symptoms^18^, Anxiety and depression were evaluated using Generalized Anxiety Disorder-7 (GAD-7) and Patient Health Questionnaire-9 (PHQ-9), respectively^19,20^. A GAD-7 score ≥ 8 and PHQ-9 score ≥ 10 indicated the presence of anxiety and depression, respectively^21,22^. FM was diagnosed according to the 2016 ACR criteria^17^. Anxiety, depression, and FM were evaluated in all participants with EM and CM, and in controls. Medication overuse (MO) was diagnosed as follows, based on the MOH criteria (code 8.2): regular intake of triptans, ergotamine, combination analgesics, and opioids for ≥ 10 days/month or regular intake of non-opioid analgesics on ≥ 15 days/month for > 3 months. For participants using multiple classes of drugs, we applied the criteria for MOH attributed to multiple drug classes not individually overused (code 8.2.6)^14^. The criterion of headache day frequency (≥ 15 days/month) was not applied in the MO diagnosis.

### Statistical analysis

Binary and ordinal variables are showed as numbers and percentages. The normality of data was assessed using the Shapiro–Wilk test. Normally and non-normally distributed continuous variables were examined using an independent t-test or analysis of variance and the Mann–Whitney or Kruskal–Wallis tests, respectively. The results of the latter tests are expressed as medians (interquartile ranges [IQR]). Categorical variables were examined using the chi-square test.

The area under the curve (AUC) for each receiver operating characteristic (ROC) curve was obtained to measure the discrimination capacity of plasma glutamate levels (control vs. EM and control vs. CM). Poisson regression analysis with adjustment for age was used to evaluate the association between the monthly headache frequency and plasma glutamate levels. Post hoc analyses were conducted using Bonferroni’s method.

We set the statistical significance at *p* < 0.05 (two-tailed). For among-group comparisons of plasma glutamate levels through post hoc analyses, statistical significance was set at *p* < 0.017 (0.050/3). Except for ROC analysis and sample power calculation, all statistical analyses were conducted using IBM SPSS software version 25 for Windows (IBM Corp., Armonk, NY, USA). ROC analysis was conducted using EzR version 1.4.5^23^. Sample power was calculated using G*Power version 3.1.9.6^24^ The analyses were planned before data collection. There were no missing data.

### Ethical approval

This study was reviewed and approved by the Institutional Review Board of Severance Hospital, Yonsei University (approval No. 2018–2711–004). This study was conducted following the principles of the Declaration of Helsinki and its subsequent amendments^25^. All participants provided written informed consent before study participation.

## Results

### Demographic and clinical characteristics of the participants

We enrolled 240 women (EM [n = 98], CM [n = 92], and control [n = 50]). Compared with the EM group, the CM group had a higher prevalence of phonophobia, depression, FM, and MO as well as a lower prevalence of unilateral pain and migraine with aura. There was no significant among-group difference in the age and body mass index (Table 1). Among the 190 participants with EM or CM, 9 and 181 participants were classified as having migraine with aura (code 1.2) and migraine without aura (code 1.1), respectively. Of the 98 participants with EM, 24 (24.5%), 19 (19.4%), and 12 (12.2%) participants had anxiety, depression, and FM, respectively. Of the 92 participants with CM, 33 (35.9%), 46 (50.0%), and 56 (60.9%) participants had anxiety, depression, and FM, respectively. None of the healthy controls had anxiety, depression, or FM.

**Table 1:** Demographic and clinical characteristics of the study participants.

	Episodic migraine, N = 98	Chronic migraine, N = 92	Controls, N = 50	*p*-value
Age, year, median and IQR	42.00 (31.00–51.00)	43.00 (33.00–51.00)	45.00 (31.75–53.25)	0.908*
Body mass index, median and IQR	21.10 (19.61–23.46)	21.73 (19.97–24.56)	21.96 (19.90–23.86)	0.245*
Headache frequency per month, median, and IQR	4.00 (3.00–8.00)	22.00 (17.25–30.00)		< 0.001^†^
**Headache intensity**				0.382^†^
Mild, N (%)	0 (0.0)	1 (1.1)		
Moderate, N (%)	14 (14.3)	9 (9.8)		
Severe, N (%)	84 (85.7)	82 (89.1)		
Unilateral pain, N (%)	60 (61.2)	30 (32.6)		< 0.001^†^
Pulsating quality, N (%)	95 (96.9)	91 (98.9)		0.622^†^
Aggravation by movement, N (%)	72 (73.5)	77 (83.7)		0.087^†^
Nausea, N (%)	91 (92.9)	86 (93.5)		0.865^†^
Vomiting, N (%)	39 (39.8)	31 (33.7)		0.384^†^
Photophobia, N (%)	43 (43.9)	44 (47.8)		0.585^†^
Phonophobia, N (%)	44 (44.9)	58 (63.0)		0.012^†^
Anxiety (GAD-7 score ≥ 8), N (%)	24 (24.5)	33 (35.9)	0 (0.0)	0.087^†^
Depression (PHQ-9 score ≥ 10), N (%)	19 (19.4)	46 (50.0)	0 (0.0)	< 0.001^†^
Fibromyalgia, N (%)	12 (12.2)	56 (60.9)	0 (0.0)	< 0.001^†^
Preventive medications, N (%)	27 (27.6)	33 (35.9)		0.218^†^
Medication overuse, N (%)	3 (3.1)	18 (19.6)		< 0.001^†^
Migraine with aura, N (%)	9 (9.0)	0 (0.0)		0.003

*GAD-7* Generalized Anxiety Disorder-7, *IQR* interquartile range, *PHQ-9* Patient Health Questionnaire-9.

*Comparison among all three groups.

^†^Comparison between participants with episodic and chronic migraine.

### Among-group comparisons of plasma glutamate levels

**Figure 1:**
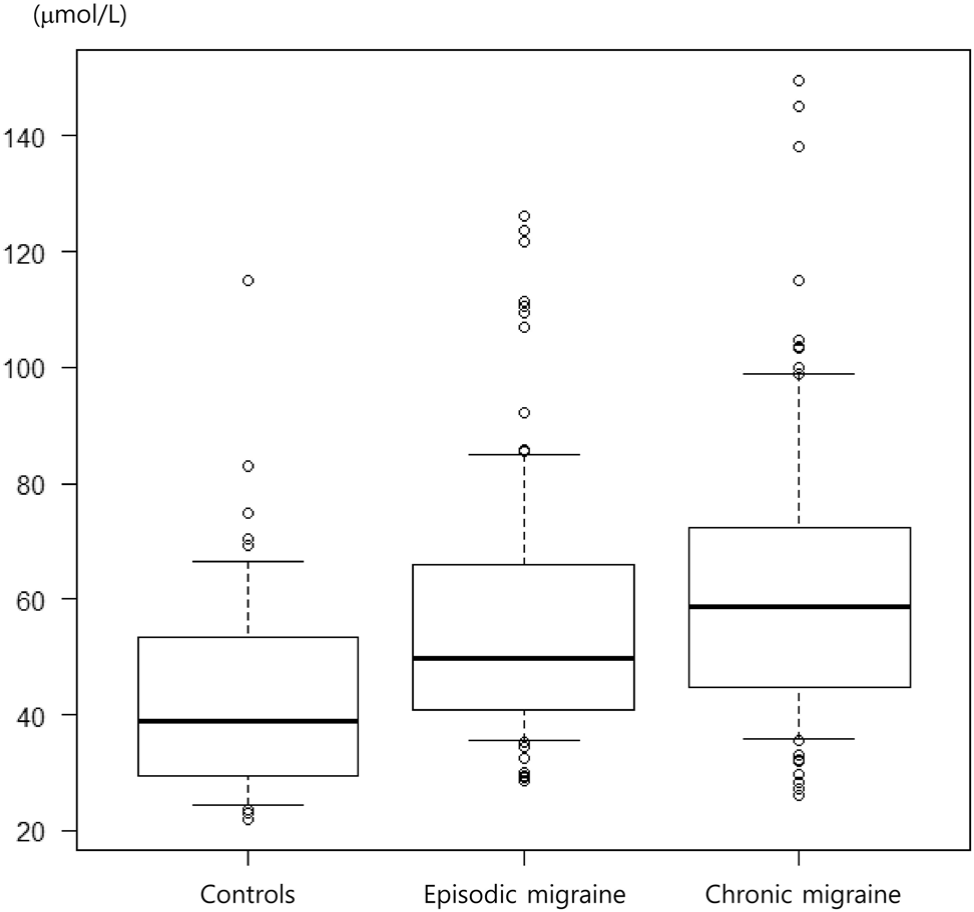
Box plot showing plasma glutamate levels in patients with episodic migraine (n = 98), chronic migraine (n = 92), and healthy controls (n = 50). In the box plots, the boundary of the box closest to zero indicates the 25th percentile. A black line within the box marks the median. The boundary of the box farthest from zero indicates the 75th percentile. Whiskers above and below the box indicate the 10th and 90th percentiles, respectively.

There were significant among-group differences in the plasma glutamate levels (EM group, 49.73 [40.82–66.12] μmol/L; CM group, 58.70 [44.64–72.46] μmol/L; control group, 38.79 [29.50–53.60] μmol/L; *p* < 0.001; Fig. 1). Post hoc analysis revealed significant differences in plasma glutamate levels between each migraine group and the control group (both *p* < 0.001) but not between the EM and CM groups (*p* = 0.075). After excluding participants with FM (n = 63) and MO (n = 20), there were significant among-group differences in the plasma glutamate levels (EM: 49.71 μmol/L, IQR: 40.38–65.56 μmol/L; CM: 56.19 μmol/L, IQR: 44.47–87.67 μmol/L; control: 38.79 μmol/L, IQR 29.50–53.60 μmol/L; *p* = 0.001). Furthermore, there were significant differences in the plasma glutamate levels between each migraine group and the control group (both *p* = 0.001). However, there was no significant difference in plasma glutamate levels between the EM and CM groups (*p* = 0.191). Plasma glutamate levels significantly differed among participants with migraine with aura (49.26 [42.23–56.06]), participants with migraine without aura (55.65 [42.15–70.37]), and healthy controls (38.80 [29.50–53.60]) (*p* < 0.001). Additionally, there were significant differences in glutamate levels between participants with migraine without aura and controls (*p* < 0.001). However, there was no significant difference in the plasma glutamate levels between participants with aura and controls (*p* = 0.055) as well as between participants with migraine with and without aura (*p* = 0.244).

### Diagnostic utility of plasma glutamate levels

**Figure 2:**
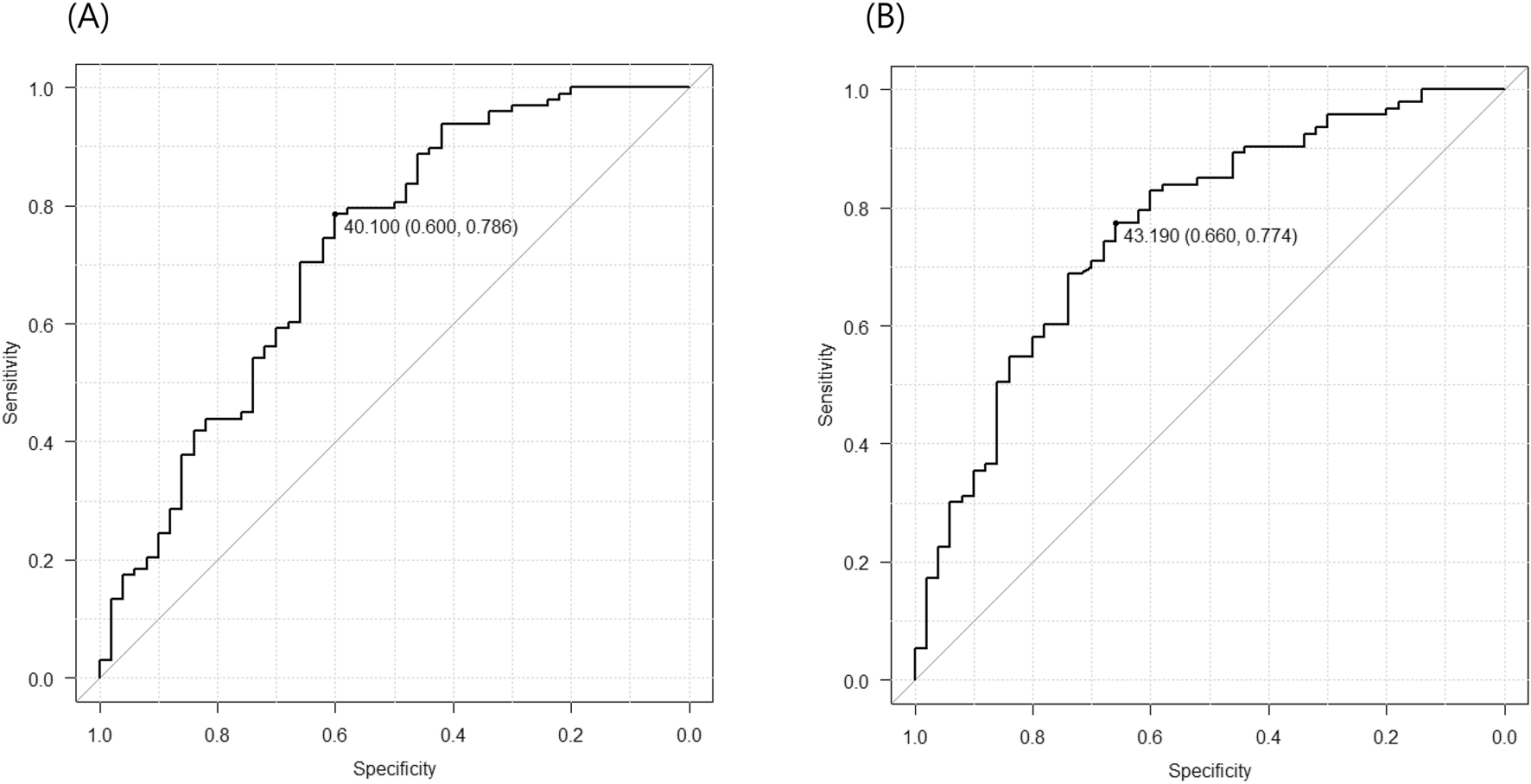
Receiver operating characteristic curves between patients with episodic migraine and healthy controls (**A**) as well as between patients with chronic migraine and healthy controls (**B**). The points for maximal area under the curve are highlighted with the sensitivity and specificity values.

The maximal Yuden index for differentiating between participants with EM and healthy controls was achieved at 40.10 μmol/L, with an AUC of 0.724 (95% bootstrap confidence interval [CI]: 0.633–0.815) (Fig. 2A). Based on this threshold, 69.6% and 78.6% of participants with EM and healthy controls, respectively, were correctly assigned.

The maximal Yuden index for differentiating between participants with CM and healthy controls was achieved at 43.19 μmol/L, with an AUC of 0.764 (95% bootstrap CI: 0.681–0.847) (Fig. 2B). Based on this threshold, 77.4% and 66.0% of participants with CM and healthy controls, respectively, were correctly assigned. The diagnostic utility at these thresholds for EM and CM including sensitivity, specificity, accuracy, positive predictive value, and negative predictive value were estimated (Table 2).

**Table 2:** The sensitivity, specificity, accuracy, positive-predictive value, and negative-predictive value for episodic and chronic migraine based on the cut-off values for plasma glutamate levels.

	Sensitivity, (%), 95% confidence interval	Specificity, (%), 95% confidence interval	Accuracy, (%), 95% confidence interval	Positive predictive value, (%), 95% confidence interval	Negative predictive value, (%), 95% confidence interval
Chronic migraine	77.4 (67.6–85.5)	66.0 (51.2–78.8)	73.5 (65.4–80.5)	80.9 (73.9–86.4)	61.1 (50.7–70.6)
Episodic migraine	78.6 (69.1–86.2)	60.0 (45.2–73.6)	72.3 (64.4–79.3)	79.4 (73.0–84.6)	58.8 (47.9–69.0)

### Plasma glutamate levels according to clinical characteristics and migraine comorbidities

Plasma glutamate levels according to clinical characteristics and comorbidities were comparable among 190 participants with EM and CM except for those with photophobia (Table 3). Specifically, participants with photophobia showed lower glutamate levels than participants without photophobia.

**Table 3:** Glutamate levels in participants with migraine according to clinical characteristics and comorbidities.

	With relevant characteristics, (μmol/L)	Without relevant characteristics, (μmol/L)	*p*-value	Sample power
Unilateral pain	51.32 (40.82–63.27)	59.81 (43.08–74.65)	0.070	0.457
Pulsating quality	55.45 (42.39–69.53)	42.22 (31.70–52.28)	0.089	0.348
Aggravation by movement	55.25 (42.31–69.29)	49.71 (42.31–70.00)	0.906	0.051
Nausea	55.06 (42.40–69.20)	55.25 (39.43–88.80)	0.975	0.115
Vomiting	51.56 (40.81–62.42)	59.08 (43.27–74.11)	0.096	0.397
Photophobia	51.32 (41.92–62.32)	59.95 (42.41–77.83)	0.029	0.561
Phonophobia	55.45 (40.29–68.03)	54.23 (43.48–70.95)	0.633	0.036
Anxiety (GAD-7 score ≥ 8)	49.71 (42.58–66.88)	56.19 (42.09–69.76)	0.428	0.125
Depression (PHQ-9 score ≥ 10)	55.25 (43.57–70.81)	55.06 (40.88–68.70)	0.650	0.074
Fibromyalgia	57.09 (44.04–70.44)	51.56 (41.62–69.15)	0.578	0.015
Medication overuse	62.27 (53.53–72.62)	52.76 (41.62–69.15)	0.079	0.235
Migraine with aura	49.26 (42.23–56.06)	55.65 (42.14–70.37)	0.244	0.734

*GAD-7* Generalized Anxiety Disorder-7, *PHQ-9* Patient Health Questionnaire-9.

There was no significant association between glutamate levels and the monthly headache frequency (exponential and 95% CI: 1.285 [0.941–1.755]). Furthermore, glutamate levels did not significantly differ among participants with mild (59.95 μmol/L; IQR: 59.95–59.95 μ mol/L), moderate (52.76 μmol/L; IQR: 40.83–106.89 μmol/L), or severe headache intensity (55.16 μmol/L; IQR: 42.34–68.03 μmol/L) (*p* = 0.472).

In the EM group, there was no significant association of plasma glutamate levels with monthly headache frequency (exponential and 95% CI 0.378 [0.085–1.683]) and headache intensity (moderate, 56.80 [39.82–90.35] vs. severe, 49.68 [41.17–65.45], *p* = 0.337). In the CM group, there was a non-significant association of plasma glutamate levels with the headache frequency (exponential and 95% CI, 1.388 [0.697–2.765]) and intensity (mild, 59.95 [59.95–59.95] vs. moderate, 52.76 [43.50–138.11] vs. severe, 58.71 [44.15–71.34], *p* = 0.735).

### Plasma glutamate levels according to preventive medications

Sixty participants (31.6%) with EM or CM received preventive treatments for migraine. None of the participants received anti-calcitonin gene-related peptide antibody or botulinum toxin A treatment. Plasma glutamate levels did not significantly differ according to the classes of migraine preventive medications taken by the participants (Table 4). Furthermore, plasma glutamate levels did not significantly differ according to the use of anti-glutamatergic medications (topiramate and zonisamide).

**Table 4:** Glutamate levels according to the classes of preventive medications.

	Number of participants using the corresponding class of preventive medications, N (%)	Plasma levels in users of the corresponding class of preventive medications (μmol/L)	Plasma levels in non-users of the corresponding class of preventive medications, (μmol/L)	*p*-value	Sample power
All preventive medications	60 (31.6)	55.45 (41.92–68.49)	53.91 (42.65–72.46)	0.874	0.182
Antiepileptic drugs	52 (27.4)	54.86 (41.90–70.22)	55.25 (42.40–69.28)	0.876	0.075
Antidepressants	8 (4.2)	52.04 (43.51–100.57)	55.25 (42.32–69.20)	0.583	0.102
Beta blockers	16 (8.4)	53.29 (43.74–63.02)	55.15 (42.18–69.53)	0.874	0.218
Calcium channel blockers	4 (2.1)	49.22 (43.62–53.58)	55.45 (42.34–69.53)	0.419	0.120
Anti-glutamatergic medications (topiramate and zonisamide)	51 (26.8)	53.91 (41.90–70.68)	55.25 (42.40–69.28)	0.876	0.075

## Discussion

Our study presented several major findings. First, compared with the control group, the EM and CM groups showed significantly increased plasma glutamate levels. Second, there was no significant difference in plasma glutamate levels between the EM and CM groups. Third, there was no significant association of glutamate levels with headache frequency, headache intensity, most typical headache characteristics, and preventive treatment. These findings confirm our hypothesis that plasma glutamate levels are elevated in individuals with EM and CM.

Several studies have reported increased ictal and interictal elevation of plasma glutamate^5,7–9,26,27^. However, none of the studies have evaluated plasma glutamate levels separately in participants with EM and CM^13^ or the association of clinical characteristics with glutamate levels. Our findings suggested that the interictal plasma glutamate levels may reflect the presence, rather than status, of migraine.

The mechanisms underlying the increased plasma levels in participants with EM and CM remain unclear. One possible explanation is that the increase in glutamate levels in neurons and platelets can affect plasma glutamate levels in participants with migraine. Neurons and platelets are the main sources of plasma glutamate^28,29^. Individuals with migraine show increased interictal CSF glutamate level, which suggests they have elevated glutamate levels in the central nervous system (CNS)^10,12,30,31^. Moreover, neuroimaging studies have demonstrated increased interictal glutamate levels in participants with migraine^32,33^. Although glutamate cannot pass through the blood–brain barrier (BBB), excessive neuronal glutamate shift from the CNS to plasma may occur through increased BBB permeability via activation of the N-Methyl-D-aspartic acid receptor^34^. Furthermore, glutamate transfer can occur in small CNS regions lacking the BBB, which are termed as circumventricular organs^35^. There is a close correlation between plasma and CSF glutamate levels^36^. Moreover, platelets can be a glutamate source since, similar to glutamatergic neurons, they have high-affinity glutamate transporters and contain glutamate granules^37,38^. Platelet glutamate can contribute to glutamate accretion in the brain^29^. Participants with migraine also show increased levels of platelet glutamate^26^.

In our study, the diagnostic accuracy of plasma glutamate levels for differentiating participants with EM and CM from healthy controls was moderate for EM (AUC = 0.724) and CM (AUC = 0.764)^39^. Ideally, we should select tests with high sensitivity, specificity, and accuracy; however, it is also important to consider the characteristics of the conditions being tested^40^. Most biomarker studies on migraine headaches have reported increased plasma glutamate levels^13,41^. However, none of the studies have reported the diagnostic utility of plasma glutamate levels in the diagnosis of EM and CM. Our findings could inform future biomarker studies on migraine.

Increased salivary glutamate levels have been reported in individuals with CM, but not EM^11^. In contrast, we observed no significant differences in glutamate levels between the EM and CM groups. This inconsistency could be attributed to differences in the sample sources. Alterations in glutamate levels in participants with migraine may differ across samples. For example, compared with controls, participants with migraine with and without aura have altered plasma glutamate levels^27^. However, there was no significant among-group difference in the erythrocyte glutamate levels.

There is substantial evidence indicating the role of glutamate in migraine pathogenesis. Genome-wide association studies implicate genes that are involved with glutamate signaling in migraine, and gene mutations responsible for familial hemiplegic migraine and other familial migraine syndromes may influence glutamate signaling^42,43^. Animal studies indicate that glutamate plays a key role in pain transmission, central sensitization, and cortical spreading depression^44–46^. Multiple therapies that target glutamate receptors, including magnesium, topiramate, memantine, and ketamine, have been reported to have efficacy in the treatment of migraine^47^. Glutamate contributes to endothelial dysfunction through oxidative stress and apoptosis^48^. This finding suggests a role of glutamate in the link between migraine and stroke. Our study provides additional evidence implicating glutamate in the pathogenesis of migraine, especially CM.

Despite presenting some credible evidence in this research field, our study has several limitations. First, we only enrolled women to avoid the potential effects of sex differences in glutamate levels. Therefore, our findings may not reflect glutamate levels in male participants with EM and CM. There have been inconsistent findings regarding sex differences in plasma glutamate levels. Some studies have reported significant sex differences^49^ while other studies have reported no significant differences^50,51^. If there is a difference in the levels of glutamate between women and men, the effects of sex-hormones can be considered. Since sex-hormones fluctuate during menstrual cycles, if there is a difference in the glutamate levels between women and men, the relationship between menstrual cycle and blood sampling time should be considered. Second, the present study did not include participants aged < 19 and > 65 years. Although plasma glutamate levels did not significantly differ according to age, findings in the present study did not reflect glutamate levels in that age group^52^. Therefore, it will be necessary to measure the plasma glutamate levels in different age groups. Third, although we enrolled a sufficient sample size based on a previous study, the sample sizes in some subgroup analyses might have been insufficient. Specifically, all glutamate level differences according to clinical characteristics, comorbidities, and preventive treatment did not have sufficient sample power, suggesting an insufficient sample size. Nevertheless, we presented these results to provide more information on the glutamate levels in participants with EM and CM according to various conditions. Therefore, further studies with a sufficient sample size are needed for comparison of glutamate levels among migraine participants according to these conditions. Fourth, we enrolled participants with EM and CM from a single tertiary-care university hospital. Therefore, our findings may lack generalizability. Future studies on various populations are warranted for more generalizable findings. Fourth, we did not evaluate the migraine duration and could not evaluate the relationship of disease duration with glutamate levels. Disease duration and headache frequency are associated with brain damage in individuals with migraine and may affect brain and plasma glutamate levels^53^. However, we observed no significant association of headache frequency with plasma glutamate levels. Nevertheless, there is a need for further studies to assess the association between disease duration and glutamate levels.

## Conclusions

In conclusion, we evaluated plasma glutamate levels in participants with EM and CM. Compared with the control group, the EM and CM groups showed increased plasma glutamate levels. However, there was no significant difference in glutamate levels between the EM and CM groups. There were no significant differences in the glutamate levels according to headache frequency, headache intensity, and probably according to preventive treatments, MO, and FM. Our findings demonstrate that the plasma glutamate level is a potential marker for EM and CM.

## Data Availability

The datasets used and/or analyzed during the current study available from the corresponding author on reasonable request.
